# A Case of Placenta Presentation, with Excessive Flooding, at the Seventh Month

**Published:** 1818-06

**Authors:** G. F. Edwards

**Affiliations:** Member of the Royal College of Surgeons, London, and Surgeon at Bath.


*For the London Medical and Physical Journal.*

*A Case of Placenta Presentation, with excessive Flooding, at the Seventh Month.* Communicated by Mr. G. F. Edwards, M. S. A. Member of the Royal College of Surgeons, London, and Surgeon at Bath.

ON the 15th of October, 1817, I was consulted by Mrs. B. of this city, who was between six and seven months advanced in pregnancy, with her first child. She informed me, that a constant but trifling discharge of blood had appeared *per vaginam*, for several days, accompanied with wandering pain round her bowels and loins, more particularly when she was in an erect position, or when the motion of the child was frequent and strong. She was very anxious to attain her full period of gestation; but, as I considered the symptoms such as would most probably terminate in abortion, I deemed it prudent to apprise her of such opinion, at the same time cheering her with the hope, that these symptoms might subside. In order to lessen the probability of such an event, I ordered her to keep constantly on a couch, or the outside of her bed; to avoid stimulating liquors, and heated rooms; to attend to the state of her bowels, and to take the medicine which I should prescribe, consisting of Infus. Rosæ ℥vi., 50 drops of Tinct. Opii, and ʒſs. of spirits of vitriol.

In three or four days' time I visited her again, and found that the pain had abated materially, that the discharge continued, although much less; the bowels were kept lax with small doses of Epsom salts. I requested the same plan might be pursued, giving her every encouragement the nature of such a case would admit of.

On the 30th, I was sent for, and informed that Mrs. B. was much worse: she now complained of increased pain, and a free discharge of blood, which excited considerable alarm in her mind as to the event. I assured her, that such cases were not unusual, and that she must still continue to persevere in the same precautions, to take the medicine more frequently, to compose her mind, and be persuaded that time would gently lead her to a safe and happy termination of the case, and restore her to her husband and friends.

On this night she had slept several hours; the pains were less, and the uterine discharge rather abated; bowels open; skin cool; pulse 96 in a minute.

The night of the 31st passed much the same as the preceding, and every other circumstance remained as stated on the 30th. In the night, however, of this day, (Nov. 1st.) the pains and discharge were augmented considerably; and, when she attempted to move into an erect position, a violent gust of blood immediately followed.

On the morning of the 2d, I was again hastily sent for, and, finding the flooding had increased, I requested that I might be allowed to make an examination, in order, if possible, to ascertain the cause, and to consider the best means of relief. Mrs. B. readily acquiesced; and, when I passed my finger into the vagina, in the direction of the os uteri, though I distinctly reached the uterus, and felt a very small opening, yet the whole was so obscured by clotted blood, that I was some time before I could be certain of the presentation. At length, however, I succeeded, and had no doubt on my mind that the presentation was placenta. In order to be more satisfied, I made a rotatory turn or two with my finger in the orifice of the os uteri, by which means the placenta was raised, and a violent stream of blood ensued. Considering the discharge (though making a great show) not inordinate, I explained the nature of the case to the friends of my patient, and assured her, that, in the event of a continuance of the flooding, if nature did not effect her relief, that it would then be in my power. I directed them, in case the pain should become violent, or the discharge much increased, to send for me. About nine o'clock in the evening, (though at another labour,) I was requested to attend immediately, as Mrs. B.'s life was considered in imminent danger. In about half-an-hour I was released from the case I was then at, by its terminating to my satisfaction, which soon brought me to one which promised but little in its result. On my arrival, I found the attendants and nurse in a state of alarm for the safety of Mrs. B., who was, to use their expression, flooding to death. I endeavoured to calm the hurried spirits of my suffering patient, by encouraging her to hope, that I should soon be able to accomplish her release.

The discharge by this time was very profuse, and the pain but little: knowing the nature of the case, I was assured that the preservation of Mrs. B.'s life depended upon the uterus being freed from its contents. Without any further delay, I introduced the points of two of my fingers into the uterus, and then the whole of my hand with very great difficulty into the vagina. I found the os uteri so little dilated as to admit my fingers with considerable trouble. On making the attempt to get more than two into the uterus, I felt the edge of the placenta recede, which occasioned a very considerable and continuous effusion of blood; however, knowing well the result, and the alternative, I persevered, and at length succeeded in getting my hand into the uterus, accordingly, through a violent gush of blood; and, pushing past the placenta, I felt the fingers of one hand of the fœtus: these did not suit my purpose.—I then passed my hand with caution upwards towards the fundus uteri, and there met with the feet, which I was not long in bringing into the vagina, and through the os externum. Having succeeded so far, and placed the child with its back to the pubis, I was in hopes of bringing the body, and then the arms, into the world with little trouble, and, at the seventh month, to find no difficulty with the head; but, in this supposition, I was disappointed: I was compelled to use very great extracting force, before I could disengage it. The discharge continuing after the birth of the child, which was dead, I introduced my hand a second time, in order to bring away the placenta, which I found partly detached, hanging at the entrance into the uterus, and part firmly attached near the fundus, and almost separated in two. I peeled it off from its attachment, from above downwards; succeeded in getting the whole away; found the uterus contract immediately afterwards, and the flooding in a great measure stopped.

Having accomplished every thing which art could do, and finding my patient quite exhausted by loss of blood and hurry of spirits, with a small feeble pulse and cold extremities, I ordered flannels wrung out of warm water to her feet, to swallow a little warm brandy and water, and, without being disturbed, to be made as dry as possible, in the position and place she was then in, and to be left with only the nurse. Before I left her, which was nearly two hours, she rallied.—I ordered a mixture, composed of laudanum, camphor julep, and infusion of roses, every two hours during the night.

In the morning of the 3d, I visited her very early, and found that she had slept several hours; was perfectly free from pain, and had no inordinate uterine discharge; her mind was more composed: she had passed some water, and complained of excessive soreness of the abdomen. On examination, she bore the pressure of my hand without shrinking from the touch. I desired a warm flannel sprinkled with brandy might be applied, and, by way of precaution, I sent a three-grain Calomel pill to be taken immediately, and followed by a mild cathartic draught. In the evening, my patient was much better, the medicines had operated freely, and the soreness of the bowels very much abated: urine passed with ease: gave forty drops of laudanum in a draught, with infusion of roses, camphor julep, and cordial confection.

Fourth night passed with ease, and in sleep: discharge but trifling: the breasts began to fill with milk—this I considered a very favourable symptom: continued the same plan, with trifling variations; directed the nurse to move, and thoroughly change her, giving nothing but plain gruel.—From this time, no unpleasant symptoms supervened; and, in the course of three weeks, she was perfectly well.

*Observations.*—I am justified in believing, that, in this case, nature could have effected nothing; and death inevitable, had my patient been left to time and chance. Therefore, it is of the utmost importance at all times, particularly under such circumstances, to ascertain the nature of the presentation, and then to take measures commensurate to the difficulty.

To a young beginner, I am satisfied this would, for a time, have proved a very perplexing case, and it shows the necessity (after mature deliberation) of resolute perseverance, in order to effect the object proposed. If I had listened to the solicitations of Mrs. B. and withdrawn my hand, the result might have been fatal; at any rate, the same process must have been gone through a second time, and, perhaps, with very different results. I have made it a rule not to withdraw my hand but where I was sure no time would be lost; and I would recommend, under similar circumstances, when possession is once gained of the vagina and uterus, never to quit until the object of its introduction is accomplished.

*Bath; April*, 1818.

